# Preparation and Characterization of Mechanical Properties of HAP/45S5 Bioglass Laminated Ceramic Composites via Spark Plasma Sintering

**DOI:** 10.3390/ma17225413

**Published:** 2024-11-06

**Authors:** Ye Meng, Xinge Li, Bing Yun

**Affiliations:** 1 National Demonstration Center for Experimental Materials Education, University of Science and Technology Beijing, Beijing 100083, China; yylixinge@163.com (X.L.); yunb@ustb.edu.cn (B.Y.); 2 School of Materials Science and Engineering, University of Science and Technology Beijing, Beijing 100083, China

**Keywords:** hydroxyapatite, 45S5 bioglass, laminated, spark plasma sintering, biocomposite

## Abstract

Hydroxyapatite (HAP) displays a high degree of similarity to the inorganic components that make up roughly 70% of human hard tissue, and it possesses exceptional biological activity and biocompatibility. It is currently internationally recognized as the most biologically active hard tissue implant material. However, its substandard mechanical properties have significantly limited the application of HAP in areas requiring load bearing or in the repair of large bone defects. In this study, HAP/45S5 bioglass laminated ceramic composites were consolidated using the spark plasma sintering (SPS) technique. The grain growth and phase transformation of HAP and 45S5 bioglass were examined at various sintering temperatures. The mechanical properties of the laminated composites were investigated. At 950 °C, the flexural strength and fracture work of the sintered body were (153.22 ± 7.7) MPa and (2049 ± 34) J·m^−2^, respectively. These results corresponded to the load–displacement curves and showed that the composites met the mechanical performance requirements of the support material.

## 1. Introduction

In recent years, laminated composites have been widely applied in the field of ceramics to enhance their fracture toughness and prevent the catastrophic failure of ceramic materials under external loads [1,2]. Laminated ceramic materials are produced by interleaving layers of higher toughness or lower hardness between hard and brittle ceramic layers [3,4]. This type of laminated composite is designed to prevent sudden and catastrophic fracture and damage to ceramics in the event of crack formation. When the material undergoes bending and damage within its layered structure, the cracks repeatedly encounter layer interfaces during propagation, which leads to deflection or passivation. Consequently, it is difficult to advance the crack tip smoothly in the direction of propagation, thereby enhancing the toughness of the ceramic material [5,6]. Representative composites include SiC/C [7], Si_3_N_4_/BN [8] layered composite ceramics, and alumina/metal [9] layered composites. High-performance laminated ceramics have attracted widespread attention in various fields, such as engineering structures [10], aerospace [11], military armor [2], biomedical [12], and construction [13,14]. Most natural biomaterials, such as bone/shell pearl layers and biomineralized silicon, possess excellent strength and toughness, largely attributed to their natural layered structures [15,16]. This layered structure typically consists of alternating layers of hard mineral and flexible organic matter [17]. Its strength and toughness far exceed the original level of each layer of material, offering a novel research avenue for the enhancement and reinforcement of ceramic materials. Layered materials are particularly notable in that their fracture toughness can be improved while also enhancing the strength of composite materials [18]. Thus, employing various toughening techniques to enhance the toughness of the matrix material also represents a crucial approach to improving the performance of layered ceramics [19].

Derakhshani et al. [13] investigated the effect of sintering temperature on the mechanical and biological properties of Ti/HA functionally graded materials (FGMs) prepared via the SPS method. At a sintering temperature of 1150 °C, TiO_2_ and tricalcium phosphate (TCP) phases were formed in the FGM structure, resulting in a decrease in compressive strength from 265 MPa to 167 MPa and an increase in maximum hardness from 419 HV to 894 HV. Zhao et al. [20] prepared oriented carbon fiber (OCF)-reinforced magnesium-doped hydroxyapatite ceramics using the SPS method, and these ceramics exhibited good hydrophilicity. The compressive strength of OCF/Mg-HA ceramics along the CF direction reached (129.8 ± 31.3) MPa, a 70% increase compared to that measured perpendicular to the CF direction, satisfying the requirements for human cortical bone. Mansoor et al. [21] investigated using TiO_2_/HA additive to improve the physical and mechanical properties of wollastonite CaSiO_3_ ceramics. At temperatures of 1200 °C and 1250 °C, a maximum relative density of 98% was achieved. At a high temperature of 1250 °C, maximum hardness and compressive strength values of approximately 67 MPa and 225 MPa were obtained. Bellucci et al. [22] developed the BG_Ca/Mix bioactive glass by incorporating pure, Mg-substituted, Sr-substituted, and Mg/Sr-bisubstituted TCP powders using conventional sintering techniques at a low temperature of 850 °C. The authors demonstrated that this complex supports cell adhesion and proliferation, providing a promising mechanism for differentiation into an osteoblast phenotype.

Hydroxyapatite (HAP), recognized for its outstanding biocompatibility and specific mechanical properties, has been one of the most extensively researched active biomaterials, drawing significant interest from materials researchers in recent years [23,24]. HAP closely resembles the inorganic components constituting approximately 70% of human hard tissue, encompassing calcium and phosphorus, which are crucial for human tissue, and HAP is devoid of any other harmful substances [25,26,27]. After implantation within the body, calcium and phosphorus are released from the surface of the substance due to the action of bodily fluids. These elements are subsequently absorbed by human tissue and form chemical bonds with human bone tissue, thereby facilitating the growth of new tissue [28]. Therefore, hydroxyapatite ceramics are currently recognized for their excellent biocompatibility and osteoconductivity, making them bioactive ceramic materials [29] with significant theoretical and practical application potential.

45S5 Bioglass is a biocompatible material with significant bone conductivity, osteoinductivity, and controllable biodegradability [30,31]. Hench et al. [32] first prepared 45S5 bioglass in 1969, and it is mainly composed of 45 wt.% SiO_2_, 24.5 wt.% Na_2_O, 24.5 wt.% CaO, and 6 wt.% P_2_O_5_. 45S5 Bioglass exhibits outstanding biomineralization properties and is capable of reacting with bodily fluids and tissues in vivo to produce a layer of phosphate ash, specifically carbonate hydroxyapatite (CHA), akin to the inorganic components found in healthy human bone tissue. In one study, it was found that, through interface reactions, bioglass could directly form a biolayer with bone tissue, and its strength was exceptionally high due to the formation of hydroxyapatite [33].

In recent years, there have been lots of reports exploring the combination of HAP and bioglass via methods such as the sol–gel process [34,35], electrophoretic deposition [36,37], melting cooling [38,39,40], isostatic pressing sintering [41], pressureless sintering [42,43], and SPS [44,45]. Hydroxyapatite composites prepared via the sol–gel method are mainly used as porous coatings for metal implants in orthopedic and dental fields [46]. These materials combine the exceptional mechanical properties of metals with the bioactivity of hydroxyapatite [47]. Electrophoretic deposition has been widely utilized to fabricate bioactive thin films on metal implants. The aim is to circumvent the poor mechanical properties and brittleness associated with large bioactive ceramics. By applying bioactive coatings to metal substrates, it is possible to achieve a combination of both mechanical and biological properties, including bioactivity, biocompatibility, and tissue adhesion [36]. Spark plasma sintering (SPS) technology directly applies a pulsed current to pressed powder particles, thereby heating the plasma generated by spark discharge. It leverages both thermal and field effects to achieve rapid sintering at low temperatures. Compared to traditional sintering processes such as pressureless sintering, hot pressing sintering, and hot isostatic pressing, the special sintering mechanism of SPS (which includes the internal heating of powder particles, as well as electric and magnetic field effects) imparts new structures and properties to the material. This makes SPS particularly well-suited to the preparation of specialized powder materials, including those that are spherical, amorphous, and nanostructured [48]. Dubey et al. [49] proposed that HAP begins to decompose towards β-TCP (β-tricalcium phosphate) during sintering at 1200 °C. According to reports [50], in comparison to pure HAP, composites of HAP and TCP are more prone to elicit moderate initial inflammatory responses.

In this study, HAP/45S5 bioglass laminated ceramic composites were fabricated using the spark plasma sintering (SPS) technique at lower temperatures and within a reduced timeframe. The grain growth and phase transition of HAP and 45S5 bioglass were investigated at various sintering temperatures. The trajectory and methodology of layer structure cracking in HAP/45S5 bioglass laminated ceramic composites were examined. The bending strength and fracture work were assessed, and the microstructure and phase composition were analyzed using field emission scanning electron microscopy (FESEM) and X-ray diffraction (XRD). The comprehensive mechanical properties of the HAP-45S5 composite ceramic materials were enhanced by utilizing the SPS technique, which prevented the transformation of HAP into β-TCP. Herein, new ideas and methods are proposed for the development of high-performance bone repair materials.

## 2. Materials and Methods

### 2.1. Manufacturing Process

45S5 Bioglass powder was provided by MO-SCI Corporation, Rolla, MO, USA. The chemical composition (wt.%) was 45% SiO_2_, 24.5% Na_2_O, 24.5% CaO, and 6% P_2_O_5_. The theoretical density was 2.8 g/cm^3^, the softening temperature was 550 °C, and the particle size distribution was d_50_ = 2 μm, with d_90_ < 6 μm. The HAP powder we used in this study, provided by Sigma Aldrich Co., Saint Louis, MO, USA, has the molecular formula Ca_5_(PO_4_)_3_(OH). The melting point of the HAP powder was 1670 °C, with a density of 3.14 g/cm^3^, an average particle size of less than 200 nm, and a purity of 97%. The HAP powder (10 g) was mixed with alcohol (60 mL), stirred magnetically for 2 h at a speed of 300 rpm, and dried in an oven at 70 °C for 15 h. The 45S5 bioglass powder was treated in the same manner as above.

**Figure 1:**
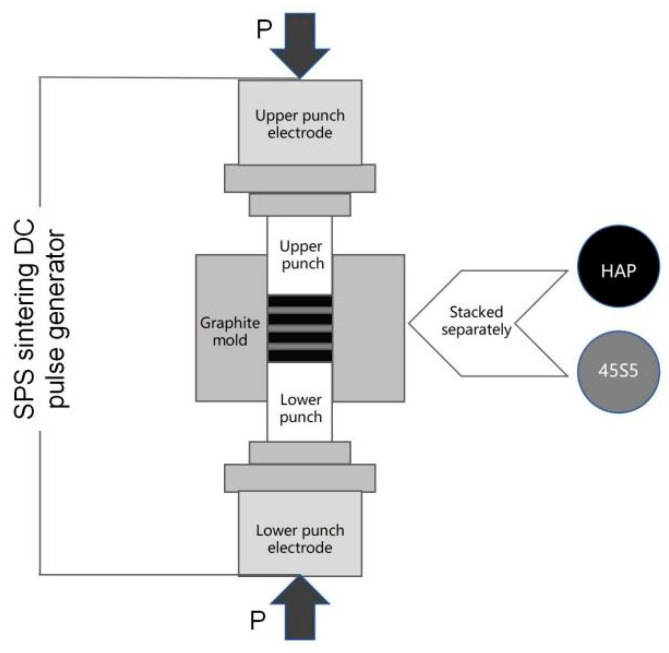
A schematic diagram of the raw material filling and sintering method for the HAP/45S5 bioglass layered composite materials.

Powders of corresponding quality were put into the graphite die layer by layer in a certain order (Figure 1). The masses of the HAP layer and the 45S5 bioglass layer used to produce the samples were 1 g and 0.5 g, respectively. The corresponding two powders were sequentially loaded into a graphite mold with an inner diameter of 20 mm. For each layer added, an axial pressure of 20 MPa was exerted on the material via a graphite indenter to ensure that the contact surfaces between the layers remained flat. The inner wall of the graphite mold and the pressure head were separated from the powder using graphite paper with a thickness of 0.2 mm to facilitate easy demolding. The preform was consolidated using SPS equipment (SPS. Dr Sinter 1050, Sumitomo Coal Mining Co., Ltd., Tokyo, Japan). A schematic diagram illustrating the raw material filling and sintering process for the HAP/45S5 bioactive glass laminated composites is presented in Figure 1. The SPS temperature range for the layered composite materials was established to be between 850 °C and 1050 °C, with an experimental point taken at every 50 °C interval. The experimental sintering parameters are shown in Table 1.

**Figure 2:**
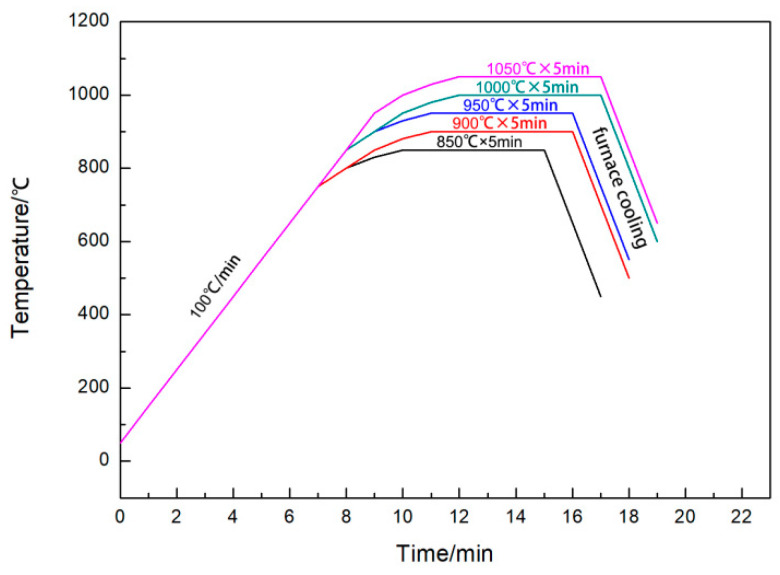
SPS heating process.

Each sample was subjected to an initial heating rate of 100 °C/min, starting at room temperature. Prior to reaching the insulation temperature of 100 °C, the heating rate was progressively decreased to 50 °C/min, then to 30 °C/min and, finally, to 20 °C/min. This approach guaranteed temperature stability during the insulation phase while enabling swift sintering, thereby preventing excessive temperature fluctuations. The heating process during sintering was depicted in Figure 2. During the sintering process, an initial pressure of 10 MPa was applied. Subsequently, as the sintering temperature increased to 500 °C, the pressure experienced an increase of 10 MPa for each 100 °C increment in temperature, ultimately culminating in a pressure level of 40 MPa. Upon completion of the heat preservation process, the pressure was gradually decreased. When the temperature descended to 500 °C, the pressure was reduced to 0 MPa. The purpose of this action was to fully alleviate the internal stresses within the material during the cooling process to prevent sample cracking.

### 2.2. Characterization

Samples were cut into 2.5 mm × 2.5 mm × 18 mm strips and mechanically polished for three-point bending experiments. The three-point bending test was measured using the Microcomputer Control Universal Test Machine (WDW-1, MTS Systems Corporation, Eden Prairie, MN, USA), along with a load sensor (STC-100KG, Vishay Precision Group, Inc., Malvern, PA, USA). The span measurement was precisely 10 mm, with a consistent loading rate of 0.5 mm/min (GB/T 4741-1999 [51]). Each data point was tested on five specimens, and the average was calculated.

The microstructures and fractured surfaces were characterized via field emission scanning electron microscopy (FESEM, Zeiss Supra55, Oberkochen, Germany). Before proceeding with observation, it was essential to coat the sample surface with carbon.

The phase analysis of the sample was performed using a Rigaku UltimaIV diffractometer (Rigaku Corp, Tokyo, Japan) equipped with a one-dimensional detector and a 48-sample automatic changer. A Cu target with λ = 1.5406 Å was used, operating at a voltage of 40 kV and a current of 40 mA, with a scanning range of 10° ≤ 2θ ≤ 100°.

## 3. Results and Discussion

### 3.1. Microstructure Analysis

**Figure 3:**
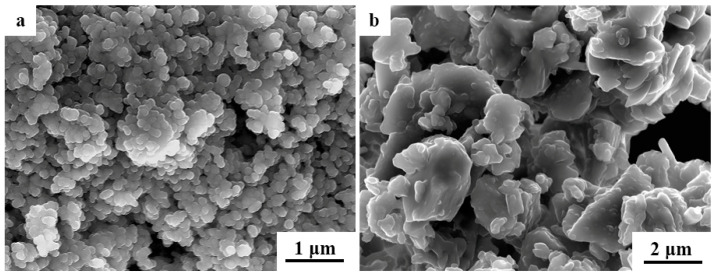
Scanning electron microscope (SEM) images of the raw materials: (**a**) pure HAP powder and (**b**) pure 45S5 bioglass powder.

In Figure 3, scanning electron microscope (SEM) images of the raw materials, hydroxyapatite (HAP) and 45S5 bioglass, are presented. The original HAP powder possessed a spherical morphology and exhibited a relatively uniform particle size distribution, with an average diameter of 200 nm (Figure 3a). The powder particles of the 45S5 bioactive glass exhibited irregular shapes and varied sizes, with larger particles (~2 μm) possessing distinct edges adhering to numerous smaller ones (<1 μm), as shown in Figure 3b.

**Figure 4:**
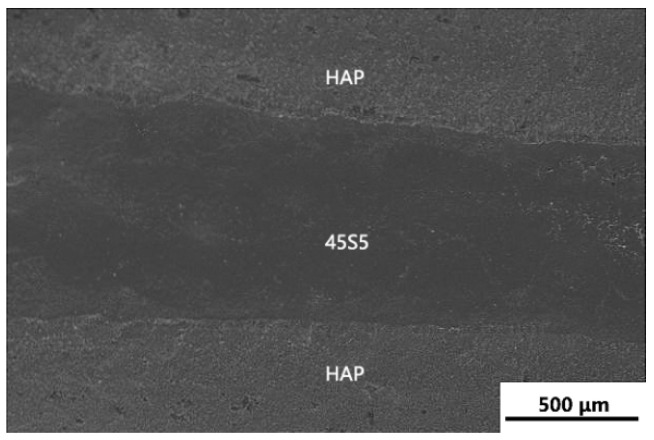
SEM micrograph of the fracture surface of the 950 °C sintered laminated HAP-45S5 bioglass ceramics.

An SEM micrograph of the fracture surface of the 950 °C sintered laminated HAP-45S5 bioglass ceramics is shown in Figure 4. This micrograph revealed a distinct parallel layered structure, with alternating gray HAP and black 45S5 layers. The 45S5 bioactive glass layer, situated between the gray HAP layers, measured approximately 800 μm in thickness.

**Figure 5:**
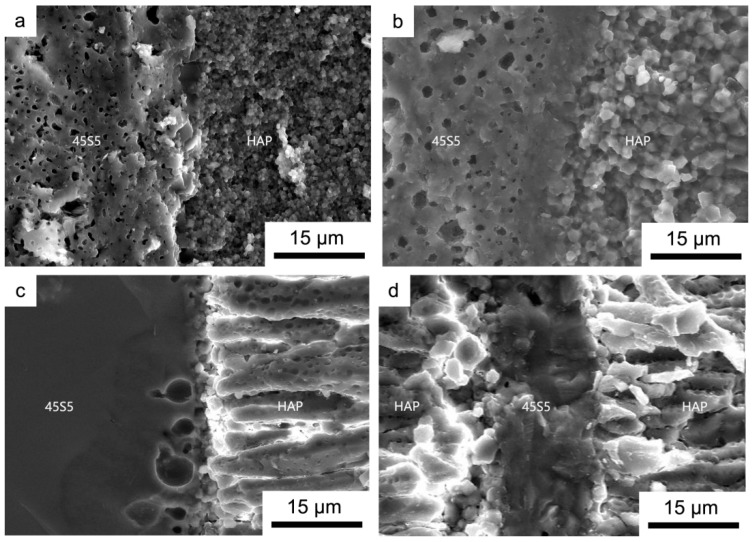
Fracture surface of laminated HAP/45S5 bioglass ceramic with different sintering temperatures: (**a**) 900 °C; (**b**) 950 °C; (**c**) 1000 °C; and (**d**) 1050 °C.

Figure 5 shows SEM images of the fracture surface of HAP/45S5 bioglass at varying sintering temperatures. Upon sintering at 900 °C (Figure 5a), the 45S5 bioglass layer exhibited a honeycomb-shaped structure with a large number of pores. The grains within the HAP layer retained a uniform spherical structure, exhibiting a consistent distribution of grain sizes, with an average diameter of 700 nm. The components connected by the two phases seemed to maintain their initial structure and morphology; however, minor cracks at the junction indicated that the interlayer composite of the laminated material’s sintered body was not entirely complete [10] at this stage.

**Figure 6:**
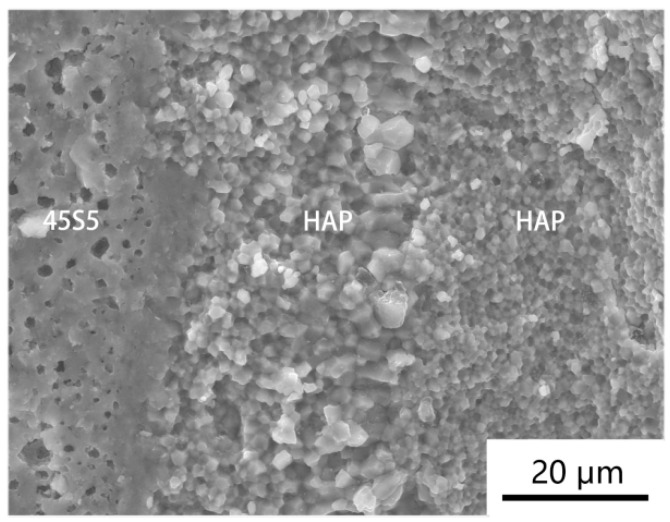
Fracture surface of HAP/45S5 bioglass layered composite material sintered at 950 °C.

Upon reaching a sintering temperature of 950 °C (Figure 5b), the 45S5 bioglass layer retained its honeycomb-shaped structure. During the sintering process, however, the holes also migrated along the grain boundaries. In this process, holes that encountered each other merged and absorbed one another, resulting in a decrease in the number of holes and an increase in their size, although the grain boundaries and morphology remained visible. However, within the region in contact with the HAP layer, the grain morphology of the 45S5 bioglass became indistinguishable, and at this interface, signs of its transition into the glass phase were already evident. The glass phase refers to an amorphous substance that forms during high-temperature sintering of ceramics [22]. This substance has the ability to bond dispersed crystal phases, inhibit grain growth, and fill pores, thereby making ceramics denser. At 950 °C, a small portion of the glass phase effectively bonded the HAP layer and the 45S5 bioglass layer and filled the existing pore, resulting in a smooth intermediate zone, which is visible in the figure below. In the neighboring HAP layer, the average particle size significantly increased to 2 μm. Conversely, in the region not depicted in Figure 5b, the growth of HAP grains further from the 45S5 bioglass layer was less pronounced, with the average particle size being only marginally larger than that at 900 °C, reaching approximately 1 μm (Figure 6).

Upon reaching a sintering temperature of 1000 °C (Figure 5c), it became apparent that the 45S5 bioglass layer had fully transformed into an amorphous glass phase. The presence of a minor quantity of glass phase within the sintered body contributes to its density; in this experiment, this was advantageous for the two-phase composite (as depicted in Figure 5b). Nevertheless, the mechanical strength of the glass phase was inherently low, and its reducibility was inadequate. The creation of such a substantial quantity of glass phase would inevitably have detrimental effects on the performance of layered composite materials. The HAP layer experienced continuous grain growth along the c-axis in a high-temperature sintered state, resulting in needle-like or columnar structures.

At a sintering temperature of 1050 °C (Figure 5d), the bioactive glass layer attained a softened condition as a result of being subjected to excessively elevated sintering temperatures. Due to the continuous application of axial pressure by the dies at both ends during the sintering process, a significant amount of softened glass phase was extruded along the mold sidewalls as a result of the increased fluidity of the glass phase. This process resulted in the remaining 45S5 glass layer thickness being reduced to just 15 μm, in contrast to the bioglass layer thickness in other sets of experiments, which was maintained at 80 μm. When the glass layer was nearly completely expelled from the sintered body, it was challenging to regard it as a structure embodying the defining characteristics of a laminated composite material.

Based on our microscopic analysis of the fracture surfaces of sintered bodies at various sintering temperatures, it can be concluded that the composite effects regarding the microstructures and interlayer bonds of the sintered bodies were the most ideal at 950 °C.

### 3.2. The Impact of the Layered Structure on Performance

#### 3.2.1. Phase Analysis

**Figure 7:**
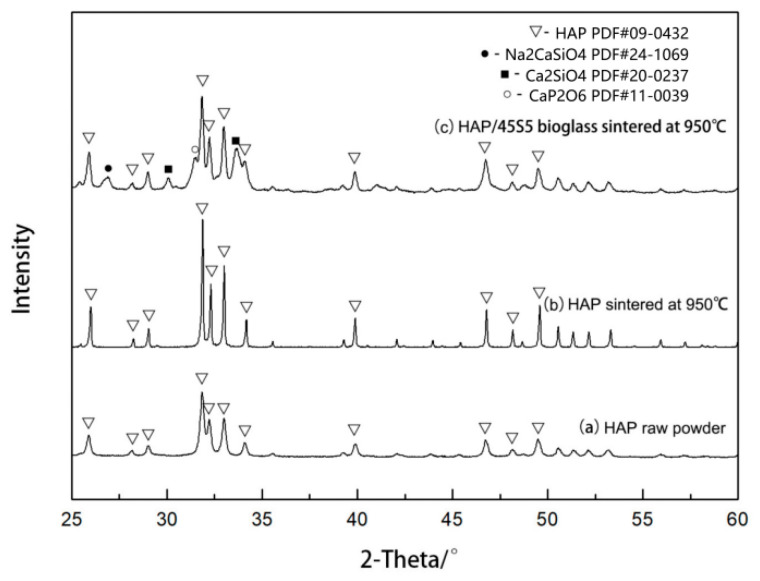
XRD patterns before and after sintering at 950 °C.

**Figure 8:**
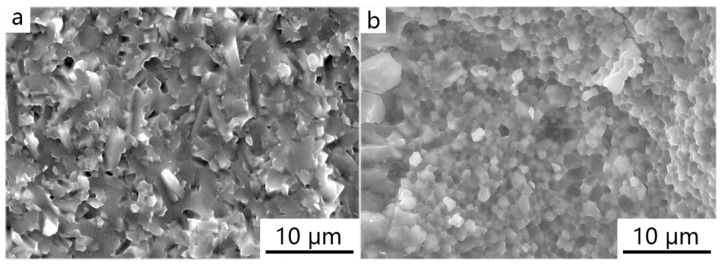
Fracture morphology of ceramic materials at 950 °C sintering temperature: (**a**) pure HAP powder; (**b**) HAP fine grain layer in HAP/45S5 bioglass layered composite materials.

The X-ray diffraction results of the original HAP powder, the pure HAP powder sintered at 950 °C, and the layered composite material of the HAP/45S5 bioglass sintered at the same temperature are compared in Figure 7. From the comparison results, it was evident that, compared to the layered composite material sintered at 950 °C, the pure HAP sintered body exhibited a narrow and sharp peak, indicating that the presence of the 45S5 bioglass layer inhibited the growth of HAP grains [52]. From Figure 8, it is evident that under identical sintering conditions of 950 °C, the grains of the pure HAP sintered body started to grow rapidly along the c-axis, forming needle-shaped and columnar crystals. In contrast, the majority of HAP grains within the sintered body of the HAP/45S5 bioglass layered composite material remained spherical, with a particle size of approximately 1 μm. Only in the region adjacent to the 45S5 bioglass layer did the HAP grains develop into particles with a diameter of approximately 2 μm, preserving a spherical morphology.

#### 3.2.2. Load-Displacement Curve Analysis

**Figure 9:**
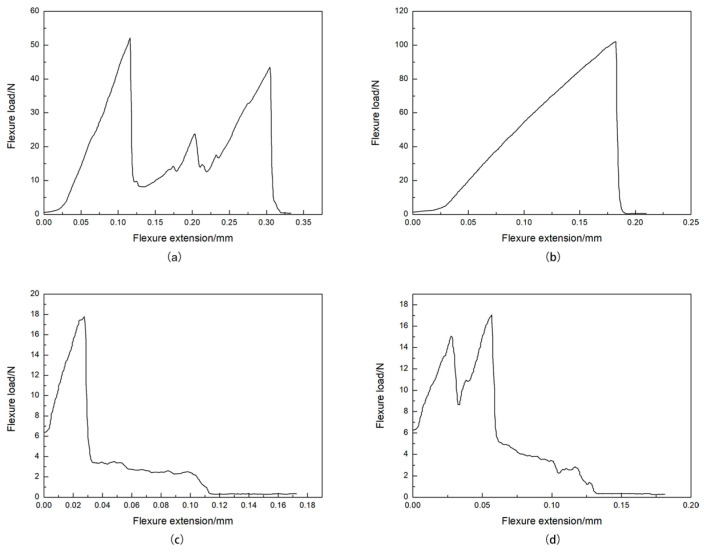
Load-displacement curves of the HAP/45S5 bioglass laminated ceramics at different sintering temperatures: (**a**) 900 °C; (**b**) 950 °C; (**c**) 1000 °C; and (**d**) 1050 °C.

Figure 9 depicts load-displacement curves derived from our flexural strength testing of the HAP/45S5 bioglass layered composite material. It was evident that at a sintering temperature of 900 °C (Figure 9a), as the displacement increased, three distinct high-load values emerged within the sintered body. At this time, the bonding between HAP and 45S5 bioglass was not sufficiently tight, and there were numerous cracks within the bonding layer. Additionally, the 45S5 bioglass layer itself was not densely sintered and exhibited a porous, honeycomb-like structure. Therefore, when an HAP layer was fractured under a higher load, the strength of the 45S5 bioglass layer diminished, and the load exerted on the weak interface decreased rapidly. When the crack propagated to the next HAP layer, the load increased as it encountered the strong interface once again, and so forth. The curve of the sintered body at 950 °C (Figure 9b) exhibited an I-shaped fracture characteristic. This was attributed to the tight interlayer bonding of the sample at this sintering temperature, leading to a sudden overall fracture. Upon reaching a sintering temperature of 1000 °C (Figure 9c), as a result of the transformation of 45S5 bioglass into its glass phase, its mechanical strength was at its lowest, and cracks propagated catastrophically as well. Therefore, when the load and displacement reached a certain threshold, the glass phases in each layer fractured almost simultaneously, resulting in a rapid decrease in load, which then stabilized at a lower level until the material fractured entirely. Upon reaching a sintering temperature of 1050 °C (Figure 9d), the overflow of the majority of the glass-phase 45S5 within the sintered body resulted in a bioglass layer with an approximate thickness of 15 μm, sandwiched between the HAP layers (Figure 9d). This configuration provided the material a resistance to crack propagation akin to that observed in the sintered body at 900 °C.

Due to the introduction of soft-interface 45S5 bioglass in the HAP ceramic materials, the uniformity within the material was modified. The introduction of 45S5 bioglass introduces more pathways for crack propagation and energy absorption mechanisms to the material.

**Figure 10:**
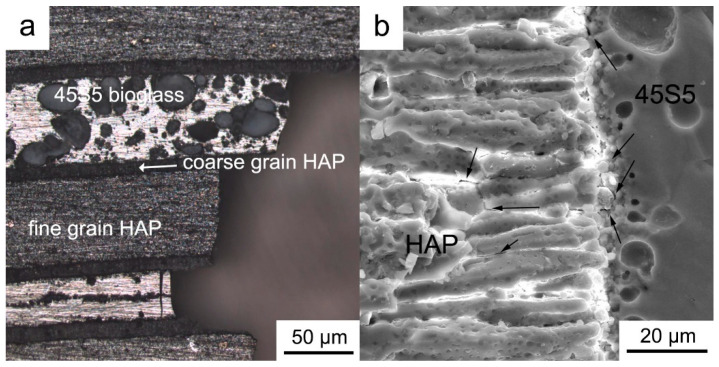
The fracture surface of the flexural sample profiles in the HAP/45S5 laminated ceramics at 1000 °C: (**a**) profile morphology; (**b**) crack.

Based on the side profile of the fracture of the HAP/45S5 laminated composite bending specimen depicted in Figure 10a, the crack propagation path within the specimen was observed. Interlayer cracks mainly occurred at the interface between the 45S5 bioglass and the HAP coarse-grained layer, indicating that the bonding strength between the layers of 45S5 bioglass and HAP coarse grains was insufficient, making cracks more likely to propagate along these weak areas. At the same time, during the propagation process, cracks frequently encountered obstacles at the layer interface, leading to deflection or passivation. This made it challenging for the crack tip to advance smoothly along the propagation direction, thereby enhancing the toughness of the ceramic materials [53]. Cracks deflected along grain boundaries after extending into the layers from the interfaces, rendering grain boundaries the primary pathway for crack propagation. In regions where the HAP grains were relatively coarse, some cracks no longer propagated solely along grain boundaries, instead exhibiting transgranular fracture phenomena. Transgranular fracture refers to a crack that passes directly through the interior of a grain, rather than propagating along the boundaries between grains. This type of fracture typically occurs in materials with larger grain sizes and internal stress concentrations. It indicates that the material has poor toughness in these areas and is prone to brittle fracture. In Figure 10b, the cracks in the material are indicated by arrows.

#### 3.2.3. Analysis of Mechanical Properties

**Figure 11:**
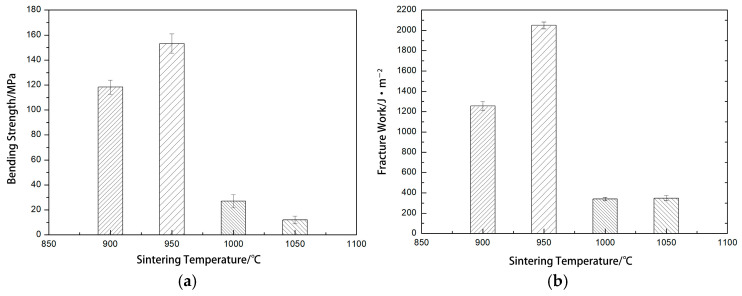
The bending strength and fracture work of laminated HAP/45S5 bioglass ceramics: (**a**) bending strength; (**b**) fracture work.

Table 2 presents the average values and standard deviations of flexural strength and fracture work for HAP/45S5 bioglass composites. Figure 11 illustrates results regarding the flexural strength and fracture work of HAP/45S5 bioactive glass laminated composites across various temperatures. As can be seen from Figure 11, the bending strength and fracture work of the sintered body were both highest at 950 °C, being (153.22 ± 7.7) MPa and (2049 ± 34) J·m^−2^, respectively, which corresponds to the load-displacement curves. An increase in fracture toughness can be influenced by several factors: ① deflection of the crack path during crack propagation; ② pinning effect of local compressive stress fields produced by secondary phases on crack growth; ③ bridge action of indehiscent ligaments on cracks (fiber enhancement); ④ energy absorption via the appearance of microcracks around the main crack; and ⑤ squeezing of the crack surfaces, which means that phase transformation expansion [54] requires more energy for cracking to proceed.

At 950 °C, the interlayer bonding of the sintered body was robust, with a small amount of glass phase adhering the HAP layer to the 45S5 bioactive glass layer and filling the pores within them. This mixed phase created a strong interface, thereby conferring high flexural strength and fracture toughness. The fracture work of the sintered body at 1050 °C increased due to the overflow of the glass phase, which led to the sintered body having a smaller cross-sectional area, resulting in a larger amount of energy required per unit surface area.

According to the report in [55], the bending strength of cortical bone (100~200 MPa) and the fracture work (2.2~2.4 kJ·m^−2^) indicates that the 950 °C sintered body met the mechanical property requirements for both cortical bone and composite bone filling materials [56,57]. Meanwhile, the presence of 45S5 bioglass modulated the dissolution of the final system to improve its biological response [58]. The 45S5 exhibited a trend towards enhanced bone-binding growth in comparison to the HAP implant, while preserving its mechanical integrity [59]. Therefore, the HAP/45S5 bioglass layered composite holds great promise as a filling material for bone defects.

## 4. Conclusions

The HAP/45S5 bioglass laminated ceramic composite was successfully prepared by using the prefabricated stacking method. In this composite, the presence of 45S5 bioglass significantly hindered the growth of HAP grains. At a sintering temperature of 950 °C, the hydroxyapatite (HAP) grains on the surface of the 45S5 layer exhibited a pronounced growth phenomenon, reaching a particle size of approximately 2 μm. At the same time, the 45S5 bioglass in contact with the HAP grains gradually transformed into a glass phase. However, in regions far from HAP, the 45S5 bioglass layer maintained its original shape and pore structure. The research revealed that fractures predominantly emerge at the boundary between the 45S5 bioactive glass layer and the HAP coarse layer. As cracks propagate, they encounter the barrier at the layer interface, resulting in crack deflection or blunting, which augments the material’s toughness. Additionally, in this study, the cracks exhibited transgranular fracture behavior in the regions characterized by coarse HAP grains. It was determined that the bending strength and fracture work peaked at 950 °C, reaching respective values of (153.22 ± 7.7) MPa and (2049 ± 34) J·m^−2^. This result was consistent with the load-displacement curves and met the mechanical property criteria for the supporting material. This indicates that the HAP/45S5 bioactive glass laminated composites demonstrate advantageous mechanical properties at 950 °C, meeting the mechanical performance standards for both cortical and composite bone graft materials. Consequently, the composites studied herein hold great potential for use as scaffold material.

## Figures and Tables

**Table 1:** Sintering parameters of HAP/45S5 bioglass laminated composites.

Temperature/°C	Pressure/MPa	Soaking Time/min	Heating Rate/°C·min^−1^	Vacuum Degree/Pa
850–1050	40	5	100	15

**Table 2:** Mechanical properties of laminated HAP/45S5 bioglass ceramics.

Serial Number	Sinter Temperature /°C	Flexure Strength /MPa	Fracture Work /J·m^−2^
F1	900	118.28 ± 5.6	1255 ± 46
F2	950	153.22 ± 7.7	2049 ± 34
F3	1000	26.9 ± 5.2	340.58 ± 18
F4	1050	11.9 ± 3.1	347.62 ± 26

## Data Availability

The original contributions presented in the study are included in the article, further inquiries can be directed to the corresponding author.
